# A MEDITERRANEAN LIFESTYLE AND FRAILTY INCIDENCE IN OLDER ADULTS: THE SENIORS-ENRICA-1 COHORT

**DOI:** 10.1093/geroni/igad104.0714

**Published:** 2023-12-21

**Authors:** Mercedes Sotos Prieto, Javier Maroto-Rodriguez, Mario Delgado-Velandía, Rosario Ortola, Fernando Rodriguez-Artalejo

**Affiliations:** University Autonomous of Madrid, Madrid, Madrid, Spain; University Autonomous of Madrid, Madrid, Madrid, Spain; University Autonomous of Madrid, Madrid, Madrid, Spain; University Autonomous of Madrid, Madrid, Madrid, Spain; Universidad Autónoma de Madrid, Spain, Madrid, Madrid, Spain

## Abstract

**Background:**

Frailty is a geriatric syndrome that entails high risk of hospitalization, disability, and death. While adherence to Mediterranean diet has been associated with lower risk of frailty, the joint effect of diet and lifestyle is uncertain. This study examined the association between a Mediterranean lifestyle (diet, customs, and traditions) and frailty incidence in older adults.

**Methods:**

We analyzed data from 1 880 individuals aged ≥ 60 from the prospective Seniors-ENRICA-1 cohort. Adherence to the Mediterranean lifestyle was assessed at baseline with the 27-item MEDLIFE index (higher scores representing better adherence), divided into 3 blocks: (1) “Mediterranean food consumption,” (2) “Mediterranean dietary habits” (practices around meals),” and (3) “Physical activity, rest, social habits and conviviality.” Frailty was ascertained as the presence of ≥ 3 of the 5 Fried criteria: (a) Exhaustion; (b) Muscle weakness; (c) Low physical activity; (d) Slow walking speed; and (e) Unintentional weight loss. Main statistical analyses were performed using logistic regression models, adjusting for the main confounders.

**Results:**

After a 3.3-year follow-up, 136 incident frailty cases were ascertained. Compared with participants in the lowest tertile of the MEDLIFE score, the OR (95% CI) for frailty was 0.88 (0.58-1.34) for the second tertile, and 0.38 (0.21-0.69) for the third tertile (p-trend = .003). Blocks 1 and 3 of the MEDLIFE score were independently associated with lower frailty risk. Most items within these blocks showed a tendency to reduced frailty.

**Conclusions:**

Higher adherence to a Mediterranean lifestyle was associated with lower risk of frailty.

